# Supplementary material for: Safety and Efficacy of Intermittent High-Dose Liposomal Amphotericin B Antifungal Prophylaxis in Haemato-Oncology: An Eight-Year Single-Centre Experience and Review of the Literature
Source: J Fungi (Basel). 2020 Dec 21;6(4):385. doi: 10.3390/jof6040385 (PMC7767522; doi:10.3390/jof6040385)
Supplement: Supplementary file 1 [file jof-06-00385-s001.pdf]

Supplementary Table S1: Mould-active† triazole drug-drug interactions and additive toxicities commonly encountered in haemato-oncology [1–4]				
**Chemotherapy /novel agent**	**Severity[4]**	**Pharmacokinetic effects**	**Toxicity**	**Recommendations**[4]‡
Anthracyclines (Daunorubicin, Doxorubicin)	Moderate		Cardiac toxicity/possible QT interval prolongation.	QT risk recommendations *
Arsenic trioxide	Severe		QT interval prolongation common with Arsenic trioxide and can last 8 weeks after treatment	Avoid azoles during/8 weeks after arsenic treatment. QT risk recommendations *
BCL-2 inhibitor (Venetoclax)	Severe	Azole CYP3A4 inhibition increases exposure to venetoclax 6–9 fold	Tumour lysis syndrome if azole administered during dose-titration phase of venetoclax.	Avoid azoles during dose-titration phase of venetoclax. Thereafter reduce venetoclax dose by 50–75%. Monitor closely for signs of toxicities.
BTK inhibitor (Ibrutinib)	Severe	Azole CYP3A4 inhibition increases exposure to ibrutinib (CMax/AUC) up to 29/24-fold respectively.	QT interval prolongation	Avoid co-administration. If given reduce ibrutinib dose to 140 mg. QT risk recommendations *
Busulfan	Severe	Itraconazole decreases clearance of busulfan by approximately 20%.	Busulfan toxicity e.g., cytopenia, gastro-intestinal (GI), mouth sores, hair loss, rash.	Avoid co-administration with high-dose busulfan. If given, monitor for busulfan toxicity. Weekly full blood counts.
Cyclophosphamide & Ifosphamide	Moderate	Azoles may inhibit the metabolism of cyclophosphamide and ifosphamide into their active form (reducing efficacy) and affect metabolism of toxic degradation products (increasing toxicity).	Cyclophosphamide/ifosphamide toxicity and/or therapeutic failure.	Monitor for cyclophosphamide efficacy as well as increases in serum bilirubin and creatinine.

FLT3 inhibitors (e.g. Midostaurin, Sorafenib, Gilteritinib)	Severe	Azole CYP3A4 inhibition increases exposure to midosaurin (1.4 fold), gileritib (1.2–2.2 fold) and sorafenib (minor).	Liver function test (LFT) derangement. QT interval prolongation	Avoid co-administration. Monitor LFTs. QT risk recommendations *
Gemtuzumab ozogamicin (Mylotarg)	Unlikel y		Potential additive hepatotoxicity	Co-administration avoided for 5 days after Mylotarg as per recommendations from the AML19 Trial. [5] Monitor LFTs.
Inotuzumab ozogamicin (Besponsa)	Mild		QT interval prolongation	QT risk recommendations *
Isocitrate dehydrogenase 1/2 inhibitors (Ivosidenib)	Not graded	Azoles CYP3A4 inhibition increases exposure to ivosidinib. Ivosidinib may reduce exposure to azoles.	Antifungal therapy failure. QT interval prolongation	Avoid co-administration. QT risk recommendations *
Vinca alkaloids (e.g., vincristine)	Severe	Azoles may increase serum vinca alkaloid concentrations	Neurotoxicity and blood dyscrasias	Avoid co-administration. If given, closely monitor for toxicity.
**Other common drugs**				
Warfarin & novel oral anticoagulants (NOACs); Apixaban, Dabigatran, Edoxaban, Rivaroxaban.	Some Severe	Azole CYP3A4 inhibition increases exposure to anticoagulants. Itraconazole p-glycoprotein (P-gp) inhibition further increases NOAC exposure. Voriconazole CYP2C9 inhibition increases	Increased risk of bleeding	Avoid azole co-administration with Rivaroxaban. Avoid itraconazole with Apixiban/Dabigatran and dose reduce with Edoxaban. Monitor INR closely and adjust warfarin on starting/stopping azole (especially voriconazole). Monitor for bleeding/anaemia. Consider age, renal function, body weight, liver function, reversibility and

		warfarin exposure.		indication before prescribing.
Calcineurin inhibitors (Cis) e.g Tacrolimus, Sirolimus, ciclosporin	Severe	Azole CYP3A4 inhibition increases exposure to CIs.	Nephrotoxicity	Avoid co-administration. If given, large dose reductions needed (e.g., Tacrolimus to 1/3 original dose with Voriconazole). Close monitoring of CI levels when starting/stopping/modifying azole treatment. Monitor renal function.
Calcium channel blockers (CCBs) e.g., Felodopine, lercanidipine, nifedipine	Severe	Azole CYP3A4 inhibition increases exposure to CCBs e.g., lercanidipine plasma 8-fold/AUC 15-fold.	CCB toxicity e.g., hypotension, ankle swelling, headaches. Itraconazole and lercanidipine are both negatively inotropic.	Avoid co-administration with lercanidipine, caution with nifedipine. Monitor for toxicity.
Carbamazepine	Severe	Azole CYP3A4 inhibition may increase Carbamazepine levels. Carbamazepine induces liver enzymes decreasing azole levels.	Antifungal therapy failure. Carbamazepine toxicity e.g., nausea, vomiting, dizziness, drowsiness, ataxia, diplopia.	Avoid co-administration. Monitor azole levels, increasing the dose if necessary. Monitor carbamazepine levels and for toxicity.
Colchicine	Severe	Azole CYP3A4 (and itraconazole P-gp) inhibition increases colchicine exposure: AUC/plasma 2.9-fold/89%.	Colchicine toxicity e.g., GI, fatigue, myalgia asthenia, overactive reflexes, paraesthesia and numbness.	Avoid co-administration in patients with renal or hepatic impairment. Otherwise, reduce colchicine dose 2-fold. Monitor for colchicine toxicity.
Corticosteroids	Moderate	Azoles CYP3A4 inhibition increases corticosteroid exposure e.g., itraconazole increases AUC of	Corticosteroids increase risk of fungal infection. Corticosteroid toxicity e.g., hyperglycaemia, weight gain, acne, easy bruising, moon face.	Consider dose reduction and monitor for enhanced corticosteroid adverse effects.

		dexamethasone 3.3–3.70-fold		
Drugs affecting stomach pH e.g., proton pump inhibitors (PPIs) and histamine receptor-2 antagonists (H2 blockers)	Moderate	Drugs lowering gastric pH reduce bioavailability of itraconazole *capsules* (but not oral solution) and posaconazole oral suspension (but not gastro-resistant tablets). PPIs increase Voriconazole exposure by CYP2C19 inhibition and azole CYP3A4 inhibition increases PPI exposure.	Antifungal therapy failure. PPI toxicity e.g., GI, dry mouth, confusion, blurred vision.	Avoid co-administration of itraconazole capsule with a PPI/H2 blockers. Avoid posaconazole suspension. Dose reduce PPI with Voriconazole.
Protease inhibitors	Severe	Azole CYP3A4 inhibition may increase ritonavir-boosted darunavir (darunavir/r). Darunavir/r induces CYP2C19 and inhibits CYP3A4 affecting azoles levels.	Antifungal therapy failure. PI toxicity e.g., GI, headache, rash.	Avoid co-administration
Rifampicin	Severe	Rifampicin induces many cytochrome P450 isoenzymes including CYP3A4 markedly reducing azole levels (80–100%)	Antifungal therapy failure	Avoid co-administration during/2 weeks after rifampicin.

Statins (Atorvastatin, Simvastatin, Lovastatin, Rosuvastatin)	Some Severe	Azole CYP3A4 inhibition increases exposure to statins: simvastatin/lovastatin (15–20 fold) > atorvastatin (fold) > rosuvastatin. 3–4	Increased risk of hepatotoxicity, myopathy and rhabdomyolysis. Symptoms include muscle pain, tenderness, or weakness, malaise, fever and/or dark coloured urine.	Avoid co-administration with simvastatin/lovastatin/atorvastatin. Caution with rosuvastatin and reduce dose.

† Interaction severity and recommendations are for voriconazole/posaconazole/itraconazole which are strong CYP3A4 inhibitors. Effects may be less pronounced for fluconazole/isavuconazole which are moderate CYP3A4 inhibitors. ‡ Recommendations from the Antifungal Drug Interactions Database [3] are presented here in a simplified form amalgamating information for Voriconazole/Posaconazole/Itraconazole for education purposes. This table should not be used directly to guide prescribing practice. * Co-administration of two agents that prolong QT interval may result in additive effects that increased risk of ventricular arrhythmias including torsade de pointes and sudden death. Avoid, especially in patients with relevant cardiac history. When co-administered, patients should be warned of symptoms that could indicate the occurrence of torsade de pointes including dizziness, light-headedness, fainting, palpitations, irregular heartbeat, shortness of breath, or syncope. Monitor ECG frequently and correct any hypokalaemia/hypomagnesaemia.

References

1. Stockley, I.; Baxter, K.; Preston, C. Stockley's Drug Interactions. Available online: www.new.medicinescomplete.com (accessed on 26 July 2020).
2. Lexicomp® Drug Interactions. Available online: http://online.lexi.com (accessed on 26 July 2020).
3. Lindsay, J.; Teh, B.W.; Micklethwaite, K.; Slavin, M. Azole Antifungals and New Targeted Therapies for Hematological Malignancy. *Curr. Opin. Infect. Dis.* **2019**, *32*, 538–545, doi:10.1097/QCO.0000000000000611.
4. National Aspergillosis Centre Fungal Infection Trust Antifungal Drug Interactions Database. Available online: https://antifungalinteractions.org (accessed on 20th November 2020).
5. Nottingham University., Russell N. AML 19 Trial: Adults with Acute Myeloid Leukaemia or High-Risk Myelodysplastic Syndrome. Available online: http://www.isrctn.com/ISRCTN78449203 (accessed on 20th November 2020).
